# DNA Dynamics during Early Double-Strand Break Processing Revealed by Non-Intrusive Imaging of Living Cells

**DOI:** 10.1371/journal.pgen.1004187

**Published:** 2014-03-13

**Authors:** Hicham Saad, Franck Gallardo, Mathieu Dalvai, Nicolas Tanguy-le-Gac, David Lane, Kerstin Bystricky

**Affiliations:** 1 University of Toulouse, UPS, Toulouse, France; 2 Laboratoire de Biologie Moléculaire Eucaryote, CNRS, UMR5099, Toulouse, France; 3 Institut des Technologies Avancées en sciences du Vivant, ITAV, Toulouse, France; 4 Institut de Pharmacologie et de Biologie Structurale, IPBS, Toulouse, France; 5 Laboratoire de Microbiologie et Génétique Moléculaires, CNRS, UMR5100, Toulouse, France; Netherlands Cancer Institute, The Netherlands

## Abstract

Chromosome breakage is a major threat to genome integrity. The most accurate way to repair DNA double strand breaks (DSB) is homologous recombination (HR) with an intact copy of the broken locus. Mobility of the broken DNA has been seen to increase during the search for a donor copy. Observing chromosome dynamics during the earlier steps of HR, mainly the resection from DSB ends that generates recombinogenic single strands, requires a visualization system that does not interfere with the process, and is small relative to the few kilobases of DNA that undergo processing. Current visualization tools, based on binding of fluorescent repressor proteins to arrays of specific binding sites, have the major drawback that highly-repeated DNA and lengthy stretches of strongly bound protein can obstruct chromatin function. We have developed a new, non-intrusive method which uses protein oligomerization rather than operator multiplicity to form visible foci. By applying it to HO cleavage of the *MAT* locus on *Saccharomyces cerevisiae* chromosome III, we provide the first real-time analysis of resection in single living cells. Monitoring the dynamics of a chromatin locus next to a DSB revealed transient confinement of the damaged chromatin region during the very early steps of resection, consistent with the need to keep DNA ends in contact. Resection in a *yku70* mutant began ∼10 min earlier than in wild type, defining this as the period of commitment to homology-dependent repair. Beyond the insights into the dynamics and mechanism of resection, our new DNA-labelling and -targeting method will be widely applicable to fine-scale analysis of genome organization, dynamics and function in normal and pathological contexts.

## Author Summary

When chromosomes break, cells must repair them to avoid becoming abnormal, cancerous or dead. The most accurate repair mechanism is based on homologous recombination (HR), in which single strands generated next to the break seek an intact replica which is copied into the broken site. Changes in chromosome dynamics during the early steps that create the single strands have not been analysed owing to lack of tools allowing analysis of this process in individual living cells. We have developed a method for directly observing the resection process that prepares DNA double-strand breaks for HR. This allows us for the first time to identify just those cells where breaks are being repaired, and so to analyze the repair mechanism with a precision not attainable using current visualization systems. We have observed that the broken DNA is prepared for restoration much faster than previously thought, and that DNA movement first slows dramatically, prior to the more pronounced movement previously seen to accompany later stages of repair.

## Introduction

DNA double strand breaks (DSB) are a major threat to chromosome integrity and cell survival. Cells meet it by launching repair programs consisting of the enzymatic restoration of the DNA and of appropriate chromatin remodelling and checkpoint activation. The exposed DNA ends are protected by the Ku70-Ku80 complex (Ku complex) until a repair pathway is chosen and corresponding proteins recruited. Direct resealing of breaks by non-homologous end joining (NHEJ) is promoted by the Ku complex but is error-prone [1]. The most precise repair pathway is replacement of the broken segment with an intact copy by homologous recombination (HR), a process conserved throughout the three kingdoms of life [2], [3]. HR is initiated by DNA end processing, during which the nucleolytic activity of the Mre11-Rad50- Xrs2/Nbs1 complex (MRX/MRN) and Sae2/CTIP generates short 3′-ssDNA tails that then serve as the substrate for extensive resection by Exo1 exonuclease or Sgs1-Dna2 helicase/endonuclease [4]–[6]. RPA binds to the exposed ssDNA and acts as a recruiting platform to assemble proteins of the recombination apparatus that enables scanning of the genome for the homologous donor.

Observation of resection has relied on indirect immunofluorescence of bromodeoxyuridine-labelled DNA [6] in fixed cells or in vivo imaging of RPA, Rad51 and Rad52 proteins that accumulate on ssDNA close to DSBs (for example see [7], [8]). As a result, the role of chromatin mobility in DSB repair has been investigated almost exclusively in relation to the homology search step that follows resection [9]. Diffusive, undirected motion of chromatin is thought to suffice for inter-chromatid HR, at least on the scale of the yeast nucleus. Tracking DSB repair proteins fused to GFP-type peptides in budding yeast suggested that DSBs gather in “repair centres” containing the HR mediator, Rad52 [10], [11]. Labelling of chromosomal sites near irreparable DSBs with fluorescent repressor-operator arrays enabled tracking of their migration to the nuclear periphery [9], [12], [13] and observation that their movement was confined within 2 h of cleavage [8]. When the homologue is present but distant, as in yeast diploid G1 cells, mobilization is needed for pairing [14], [15].

Changes in chromatin mobility accompanying resection itself have received little attention. Assessing DSB processing in single cells demands that we can distinguish cells that have incurred a break and are resecting from those that have not. We therefore sought to identify cells undergoing resection by monitoring loss of a fluorescent tag inserted immediately adjacent to a DSB site. Fluorescent operator/repressor systems (FROS) available for tagging genomic loci in eucaryotes [16]–[19] were not suitable here. The repetitive nature and large size of the operator arrays can alter short-range DNA processes such as gene domain structure, intragenic looping or DNA maintenance, and can also provoke disruptive recombinational events. In addition, tightly bound LacI and TetR repressors can create fragile sites and constitute a barrier of unknown penetrability to DNA processing enzymes [20]–[23].

We have developed an alternative DNA labelling system that circumvents these drawbacks. It has enabled us to identify budding yeast cells that undergo resection following a single HO endonuclease cut. Limiting our analysis to these cells permitted realistic calculation of DNA end resection dynamics as well as measurement of the time taken to commit to HR. In addition, use of the new tool has led to discovery of a distinct phase of confinement of chromatin neighbouring the DSB early in the resection period.

## Results

### ParB-INT, a non-intrusive DNA labelling system suitable for fine-scale studies of DNA

**Figure 1:**
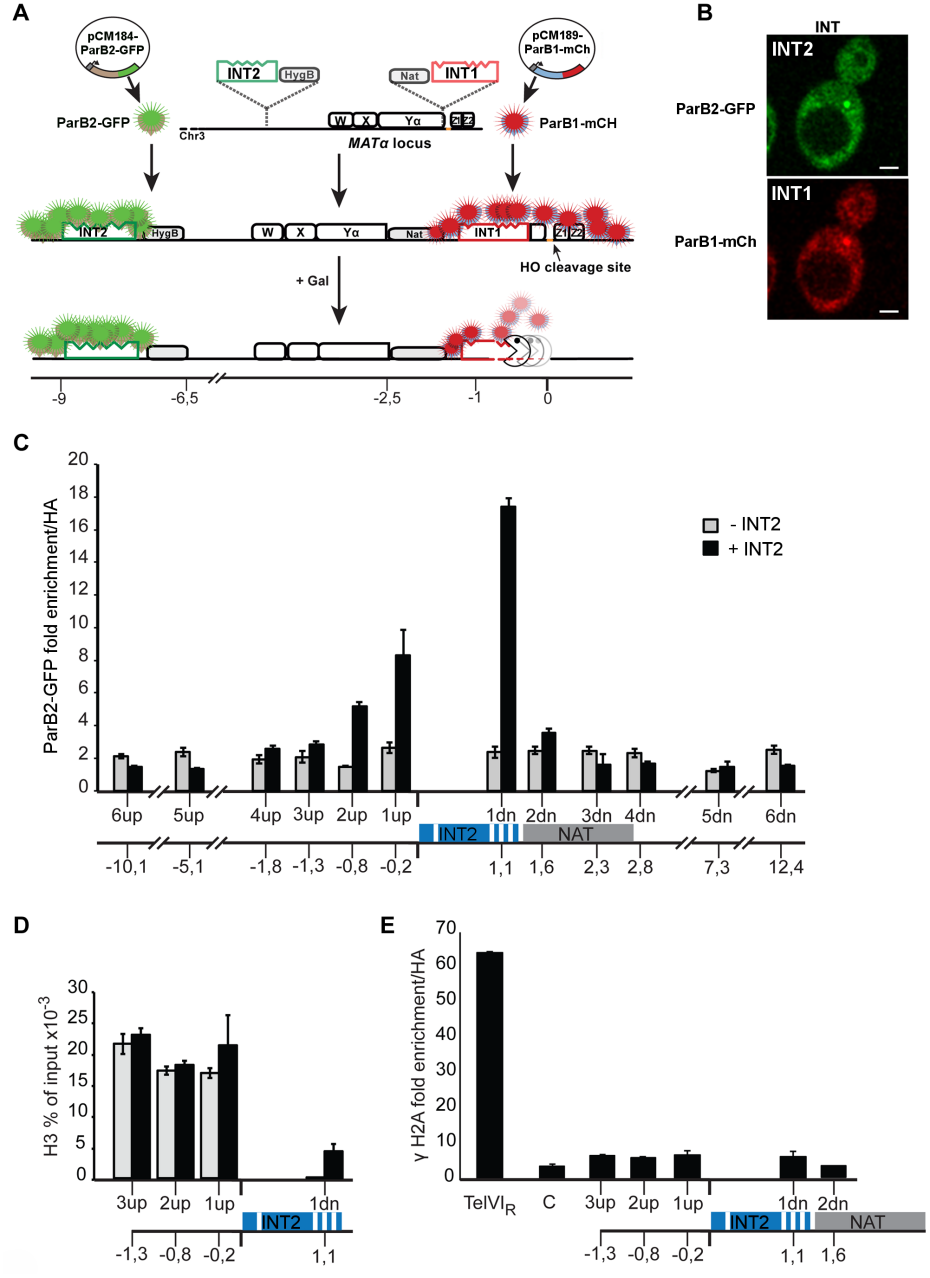
The ParB-INT DNA labelling system. **A**. INT1 and INT2, each containing four specific sites (indents) for binding their cognate ParB proteins, are inserted respectively 76 bp and 3.4 kb upstream from the HO cut-site in the *MAT* locus on yeast chromosome III. ParB1::mCherry and ParB2::GFP produced following doxycycline addition bind first to their specific sites then, through self-interaction and non-specific DNA binding, to flanking sequences, creating visible foci. Galactose addition induces HO, and the DSB it creates triggers resection which is unimpeded by the loosely-bound ParB. Disappearance of the red fluorescent spot signals passage of the resecting nuclease(s). **B**. Representative images of ParB distribution in cells labelled at INT1 and INT2. Bar = 2 µm. **C**: Spreading of ParB-GFP on chromatin flanking INT2, assayed and normalized by ChIP using anti-GFP and anti-HA respectively in strains with (black) and without (grey) INT2. **D**: Histone recruitment at and around the ParB2-INTB complex; extracts of ParB2-producing cells with and without INT2 were assayed by ChIP using anti-H3. **E**: ParB-INT does not create fragile sites. Binding of DSB marker, γH2A, was assayed by ChIP using anti-γH2A and normalized using anti-HA. Telomeric (TelVIR) and control (C, 30 kb along chromosome III) sequences serve as positive and negative controls, respectively. Experiments were performed twice. Amplicon sequences are listed in Table S2.

Our DNA-labelling tool is based on the kinetochore-like nucleoprotein complexes that activate mitotic segregation in bacteria. The protein, ParB, binds to a small (<1 kb) DNA segment that contains a cluster of *parS* sites, and then spreads along adjacent DNA. Oligomerization of fluorescent ParB, not operator multiplicity, creates fluorescent foci. The two variants used here are based on the ParB-*parS* loci of chromosomes c2 and c3 of *Burkholderia cenocepacia* J231 [24]. We have adapted this system for use in eukaryotes (Fig. 1), renaming the ∼1 kb *parS* DNA segment “INT” and the ParB proteins from the c2 and c3 chromosomes ParB1 and ParB2, respectively. Nearly all the protein is bound loosely (because non-specifically) to DNA within and flanking the INT segment and is readily displaced during transcription or repair. The ParB-INT systems do not interfere with normal growth, nor do they require host factors. These features, together with the small size (<1 kb) of the binding locus, facilitates targeted insertion into the genome and improves stability of the integrated binding sites.

To test the innocuousness of the ParB-INT system, we examined the effects of ParB2 bound to INT2 inserted near the *MAT* locus on yeast chromosome III. ParB2 associated with at least 1 kb of adjacent DNA (Fig. 1C), enough to accommodate 100–200 ParB molecules, based on the ∼20 bp occupied by each ParB dimer in bacteria [25]. The strongly reduced association of ParB with the constitutively expressed nourseothricin-resistance gene (*NAT*) suggested that transcription dominates over ParB binding; in agreement with this observation, the presence of ParB2 on INT did not reduce the level of nourseothricin-resistance and hence did not induce silencing of the neighbouring *NAT* gene (data not shown). Equal amounts of histone H3 were bound to DNA flanking the INT site in the presence and in the absence of ParB2, indicating normal nucleosome formation (Fig. 1D). Finally, γH2A was not enriched at or around INT (Fig. 1E), demonstrating that the INT insertions do not create fragile sites prone to DSB, as telomeres (Fig. 1E) and *lacO* arrays can [22].

### Analyzing resection dynamics in living *S. cerevisiae*

**Figure 2:**
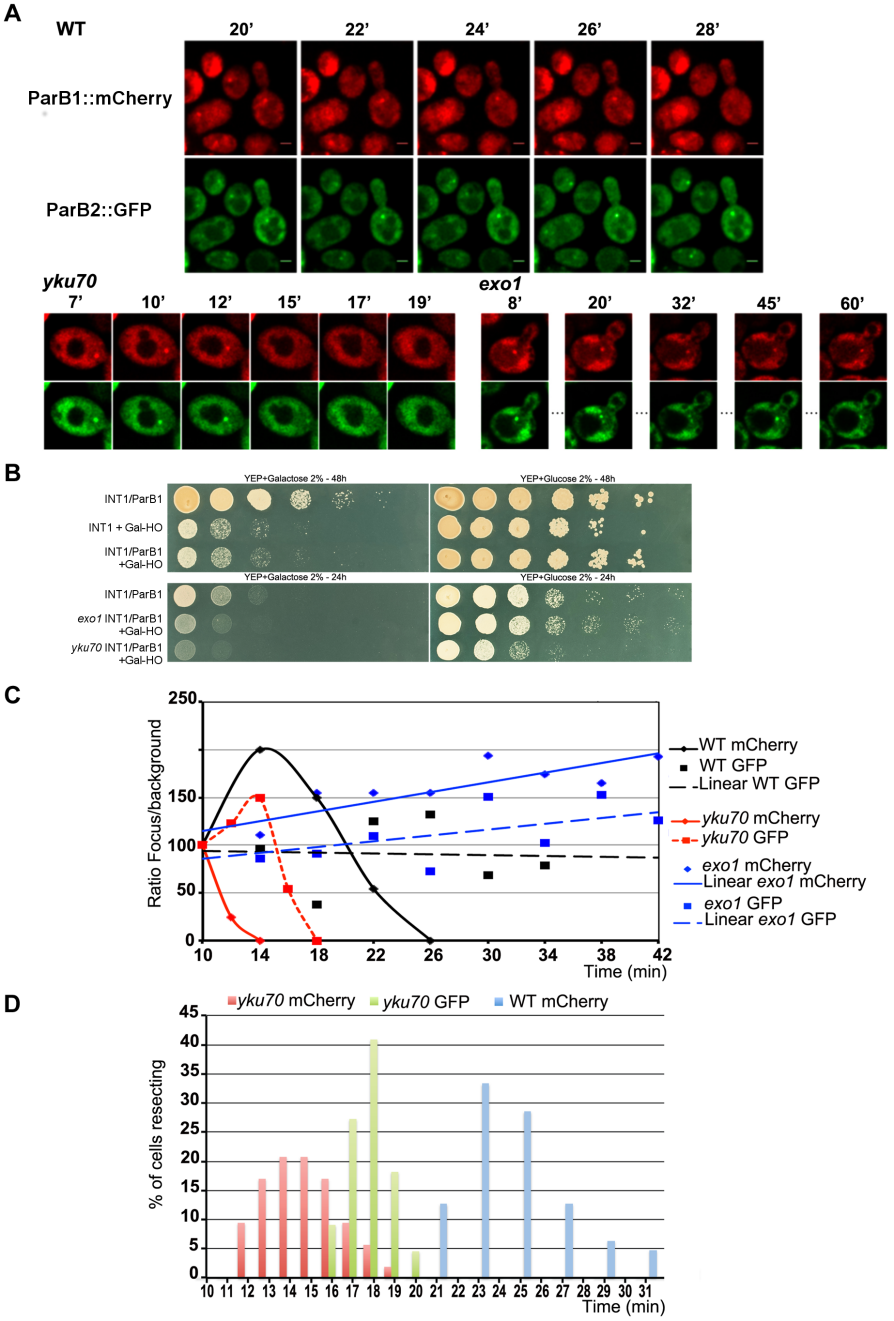
Chromosome dynamics during DNA resection in single yeast cells. **A**: Time-lapse fluorescence microscopy showing ParB1::mCherry-INT1 and ParB2::GFP-INT2 foci after induction of HO synthesis. Both signals persisted during acquisition periods of >60 min (>1500 exposures), with minimal bleaching. Representative single plane images are shown. **B**: Growth on YPE-D and YPE-Gal of wt and mutant strains bearing or not pGal-HO or ParB expressing plasmids as indicated. Cells were incubated 24 h or 48 h at 30°C and plated in 10× dilution increments. **C**: Fluorescence intensity quantification during resection in representative cells of wt, *yku70* and *exo1*strains. Intensity ratios are calculated relative to adjacent background levels. Background bleaches rapidly during initial acquisition, increasing the signal ratio. **D**: Time course of resection in wt and *yku70* cells, measured as percentage of fluorescent INT1 and INT2 foci newly disappeared at each time point. No INT2 resection was detected in wt cells within the time of the experiment.

**Figure 3:**
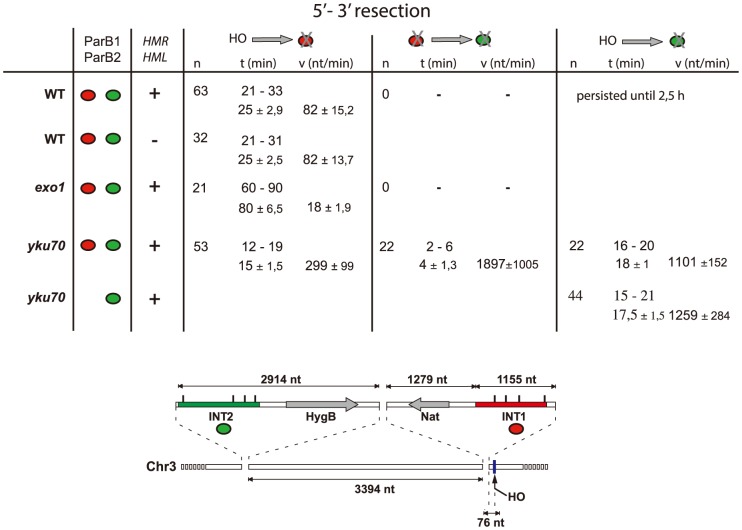
Resection timing and speed. *MAT* locus segments are resected from right to left. Data are shown as the range (top line) and average +/− standard deviation (bottom line). Calculation assumed resection to begin at the earliest time possible after HO induction (10 min), and resecting nuclease arrival at the distal end of the INT sections (1231 nt and 7612 nt from the HO cut-site for INT1 and INT2 respectively) to coincide with focus loss.

We integrated DNA fragments carrying the INT1 and INT2 variants 76 bp and 3.4 kb, respectively, from the HO cleavage site of the *MAT* locus on chromosome III of a haploid strain in which the homologous donor loci are present (Fig. 1A,B). Expression of ParB1-mCherry and ParB2-GFP generates one red and one green fluorescent INT focus that can be imaged by 3D spinning disk fluorescence microscopy in real time with minimal photobleaching [26]. To directly visualize DSB processing in living yeast cells, we monitored the two fluorescent foci after adding galactose to induce transcription of *HO* [27]. Cleavage can be detected in wt and mutant cells within 10–30 min using southern blotting (data not shown). The INT1-mCherry focus disappeared within 22–31 min from ∼60% of cells initially exhibiting both mCherry and GFP signals (Fig. 2A, WT; Video S1), while the INT2-GFP focus remained. As ParB proteins bind only dsDNA, loss of the focus indicates that the INT1 sequence has become single-stranded. Fluorescent foci were not photobleached during the time of the experiment (Video S2, S3). In our conditions, as in previous studies (data not shown; [11], [28]), a significant fraction of the *MAT* loci remains intact one hour after induction of HO synthesis, which means that accurate estimates of resection parameters can be obtained only from single cells that have incurred a break. Loss of the INT1 focus serves to identify just those cells. The cell-to-cell variability in time of cleavage by HO is highlighted by the Gaussian distribution of the time at which the INT spots disappeared (Fig. 2D). We can determine the time taken to resect the 1231 bp between the HO site and the distal end of INT1 to be 15 minutes, from the earliest time of cleavage (10 min, Figure 2D; [11]) to the earliest time of INT1 disappearance (25 min, Fig. 2D; Fig. 3). This time was the same whether or not the donor loci, *HML* and *HMR*, were present.

### The Ku complex delays resection during pathway choice

We next analysed the progress of resection in cells mutated for functions known to determine its outcome. DSBs are rapidly bound by the Ku70/80 complex, which protects the DNA ends from degradation. Ku associates with a number of proteins that prepare the ends for repair, a critical step known to delay HR [29]–[31].

In the absence of yKu70, induction of HO cleavage triggered resection of INT1 within 12–19 min (15 min average; Fig. 3; Video S4, S5, S6, S7). We can thus narrow the time taken to resect the 1231 bp between the HO site and the distal end of INT1 to 2 minutes (Fig. 2D, Fig. 3). Quantification of focus intensity in individual cells (an example is shown in Fig. 2C) illustrates the relative speed of focus loss. The intensity of the fluorescent INT1-mCherry focus in wt cells fluctuates for the first 10–12 min before it sharply declines toward extinction. We further note that resection was delayed by 10 min, on average, in wt compared with *yku70* nuclei (Fig. 2C, D). Hence the time from cleavage to onset of resection, and thus commitment to the HR repair pathway, is ∼10 min.

*In vivo* studies in budding yeast using HO-induced DSBs have shown that break repair by HR is most efficiently begun by the action of Mre11-Rad50-Xrs2 (MRX) complex and Sae2, resulting in removal of 50–100 nucleotides from the ends to create a short 3′ ssDNA tail. Normally, Exo1 nuclease or Sgs1-Dna2 helicase-nuclease then take over to carry out the bulk of the resection that generates the single strands [4], [6]. The placement of the INT1 and INT2 segments allowed us to examine this more extensive resection.

### Resection occurs in two steps

Calculation of resection rates (Fig. 3) from the moment of cleavage to loss of the INT1 signal in the wt strain yields 82 nt/min in the wt strain, a value comparable to estimates of resection speed in the literature [30], [32], [33]. Resection did not extend as far as the INT2 locus, ∼6.5 kb further on, presumably because assembly of the homology search apparatus curtailed it. In the absence of yKu however, removal of the INT1 focus was 3–4-fold faster than when measured in wt. In the absence of yKu, long-range resection nucleases may bypass MRX processing, leading to increased Exo1 activity, in agreement with previous reports [34], [35].

In 40% of the *yku70* nuclei the INT2-GFP signal was also lost. We determined that the INT2 focus disappeared within 18+/−1 min in the presence of ParB1 and 17,5+/−1,5 min in its absence, demonstrating that ParB1 did not impede DNA processing. In these cells, the GFP focus disappeared only ∼4 min after resection and loss of the INT1 label. Resection by Exo1 of the INT1/INT2 segment, which covers 8.8 kb including INT2, thus proceeded at >1200 nt/min (1900 nt/min on average). These findings directly confirm the suggestion, based on population-wide studies [4], [33], that resection is a two-step process, which undergoes a transition from an initial slow phase to a much faster one.

### Confinement of chromatin surrounding DSB during DNA end resection

**Figure 4:**
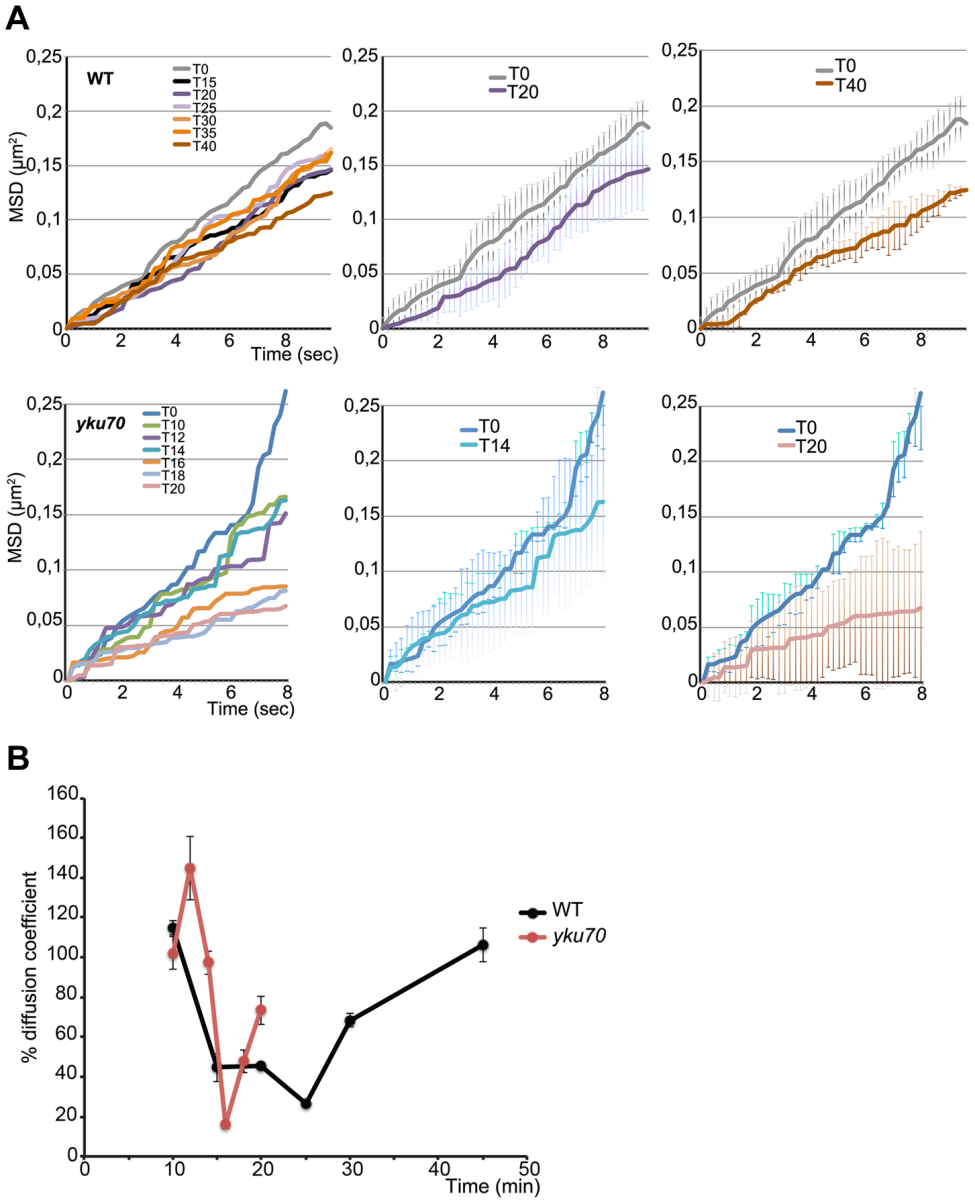
Time lapse microscopy of chromatin dynamics during resection. 2D stacks (50×200 ms frames) of the ParB2-GFP focus were acquired in wt and *yku* strains in which the ParB1-mCherry spot was lost, starting at 8 minutes post-addition of galactose to the medium. **A**. Mean-square displacement (MSD) for wt (n = 12) and *yku* (n = 3) cells of the uncleaved locus (black line) and at the indicated times after HO induction. **B**. Diffusion coefficients (D) for wt (black) and *yku* (red) calculated from the slope of the first 2 sec of the MSD shown in A and normalized to the average D = 1.9×10-2 µm2/sec for uncut DNA set at 100%.

The ability to identify cells that are resecting enabled us to record the movement of the cleaved chromatin near the *MAT* locus by tracking the INT2-GFP focus. The intact *MAT* locus moves in a freely sub-diffusive manner (Fig. 4A), consistent with previous findings [8], [36]–[38]. *MAT* mobility declined within 5 min of the disappearance of the INT1 spot from wt cells and continued to do so over the following 30 minutes. This result reveals a previously undetected loss of chromosome movement during the initial steps of DNA repair. The constraint on chromatin dynamics may reflect binding and activity of signalling and chromatin remodelling factors [39] needed for DSB processing and checkpoint activation.

To find out how this striking decline in mobility is related to resection, we measured the mobility of the INT2-GFP locus in a *yku70* mutant. MSD curves of the uncut locus showed unconstrained dynamics, followed by a decline within minutes of cleavage (Fig. 4A). The decline to minimum mobility was reached faster in *yku* than in wt cells, in keeping with the greater extent of DNA resected in the first 5 min after cleavage (Fig. 4A; Fig. 2). During 10 min of resection, the INT2-GFP locus did not recover pre-cleavage mobility, demonstrating that in the absence of yKu70, resection imposes spatial constraints on *MAT* locus dynamics.

From the initial slope of the MSD curves we also determined the diffusion coefficient (D) before and after HO induction. D of the *MAT* locus varied considerably, from 0.012 to 0.026 µm^2^/sec, as expected from its known high mobility [8], [36], [37]. Five to six minutes after cleavage, D fell steeply in both wt and *yku* cells to reach a coefficient <30% that of the uncut locus, 0.003 µm^2^/sec. Stifling the diffusion of the broken chromosome segment was thus an immediate response to DNA cleavage (Fig. 4B). After resection, D increased progressively to levels near those of the uncut locus in wt cells. Note that this recovery of D occurred 12 mins later in wt than in *yku*, reflecting the delay imposed by pathway choice (Fig. 2). The time at which normal chromosome movement resumes is consistent with the need to generate sufficient ssDNA for assembly of the HR repair machinery.

## Discussion

A new picture of the early phase of HR emerges from our findings. While numerous prior studies have identified key players in resection [40]–[46], and have outlined their successive actions and related changes in chromatin mobility [5], [33], [47], the early steps of repair following specific double-strand breakage have not been analysed at the level of single cells. One reason is the inability to directly observe the act of cleavage and the onset of resection in vivo. Despite the general utility of LacI/O- and TetR/O-based FROS in monitoring chromatin positioning and dynamics, the large size of these complexes precludes analysis of the few kilobases of resected DNA, making them inappropriate for quantitative assessment of resection. An alternative approach used to analyze HR is visualization of fluorescently-tagged proteins known to bind to the resected DNA, such as RPA, Rad51 and Rad52 [48], [49], but these suffer from some loss of normal function due to the fused fluorescent peptide and intervene only at later stages or after resection is complete.

We discovered a severe reduction in mobility of the broken chromosome ends that accompanies the initial phase of resection. The constrained DNA dynamics were not observed in earlier studies which focused on later stages of HR. Loading of repair proteins probably contributes to the rapid reduction in mobility, but cannot by itself account for the significant change in the observed diffusion coefficient. DSB ends may also interact with nuclear structures, adjacent chromatin domains or modified chromatin, possibly reflecting a need to prevent loss of contact between DNA ends prior to homology search. This would represent a security measure, as noted also by Soutoglou et al. [50]. Alternatively, the exposed ends might be held in place by a specific bridge, a role suggested for human and yeast Mre11 [51], [52]. A further alternative, that reduced dynamics might be due to an attachment to heterochromatin structures usually found near the nuclear envelope, appears unlikely in view of the retention of the cleaved *MAT* locus near the nuclear center [37]. Whatever its mechanism, the confinement of the broken ends is transient, suggesting that once the resected ends are processed and the recombination machinery is loaded, the ends now engaged in donor search resume normal chromatin motion.

Technical limitations have prevented dissection of early steps in DSB repair. The unavoidably poor synchronization of induced endonuclease (HO or I-SceI) cleavage causes a relatively wide spread in the time of initiation of resection throughout the population. The earliest assays of cleavage have typically been made 30–60 minutes post-induction, but cleavage can still be under way at 4 h, such that the cleavage rate curve is a composite of many temporally dispersed individual resections, each of which might proceed faster than the apparent rate estimated using molecular biological techniques. Our analysis of individual resecting cells shows that this is indeed the case. Thus, our analysis enabled us to determine the time from cleavage to commitment to the HR repair pathway by measuring the difference between wt and *yku* mutant cells in the time of disappearance of the first fluorescent marker, close to the DSB. During this 10 min period the Ku complex and other proteins intervene to promote ligation by NHEJ (by the Ku complex; [53]), to block resection after its initiation by MRX-Sae2 or Exo1 (by Rad9; [54]) and to activate chromatin remodelling [55]). Their activities prepare the DSB ends for repair by HR, NHEJ or other pathways.

Focussing analysis on individual cells also allowed us to determine resection speed. The speed we find for the initial resection, which includes both processing by MRX and the first ∼1 kb of resection proper, is comparable to that reported previously. It is unaffected by removal of the *HMR* and *HML* recombination target sites. Resection did not extend as far as the INT2 locus, inserted 4.6 kb from INT1, presumably because assembly of the homology search apparatus curtailed it. In the absence of yKu however, the initial phase was 3–4-fold faster than when measured in wt. Elimination of the decision phase delay rendered the observed speed, ∼300 nt/min, an accurate estimate of the combined nucleolytic activities that resect the first ∼1000 nt, although we cannot exclude the possibility that regulation of resection is perturbed in the absence of the Ku complex. Resection beyond this point is faster still, averaging nearly 1900 nt/min. In this case it can proceed as far as the distant INT2 locus, possibly because rapid onset of resection in the *yku* mutant leaves insufficient time for diversion of the resected DNA into homology search and the associated termination of nuclease activity. At least two factors could be responsible for this faster resection rate. Relatively slow MRX-mediated processing is not involved, so that digestion of the INT1–INT2 span results solely from Exo1 activity; and either a natural paucity of nucleosomes or nucleosome removal due to DSB-induced triggering of histone modification and check-point pathways might allow Exo1 obstacle-free progress along the *MAT* locus DNA. The comparable resection rates seen in the presence and absence of INT1 demonstrate that the ParB proteins themselves do not constitute a barrier to progress of the resecting nuclease. The fast rate is also comparable to that of in vitro Exo1 nucleolysis, suggesting that efficient removal of nucleosomes and other bound proteins facilitates Exo1 progress [56], [57].

An unexpected finding was the marked preference for Exo1 as the resection exonuclease, at least in our experimental set up. Previous studies suggested the Sgs1-Dna2 helicase-nuclease pair as an alternative to Exo1 during the fast, extensive resection step [33], [58]. The small fraction of *exo1* cells seen to resect suggests that Sgs1-Dna2 may be more dependent than Exo1 on initial processing by MRX-Sae2 [34] [6]; indeed the MRX complex has been seen to interact directly with Sgs1 in vitro and to stimulate Sgs1-unwinding [58], [59]. Preliminary results from our laboratory (data not shown) indicate that resection dynamics in a *mre11* mutant are similar to those in wild type, suggesting that MRX-Sae2 is dispensable for resection from HO breaks at *MAT* and that as a consequence Sgs1-Dna2 is less readily employed than Exo1 for extensive resection. It is possible in principle that Sgs1-Dna2 progress is more easily blocked by the ParB1-INT1 complex than is Exo1, although the insensitivity to INT1 in wt cells (Fig. 3) makes this an unlikely explanation. Another possible source of the apparent discrepancy between our observation and reported evidence for Sgs1-Dna2 involvement is the extended time-scale of previous resection studies, which resulted from prolonged homology search in the absence of donor loci. In our experiments, with donor loci present, resection was over less than 40 minutes after cleavage induction. Possibly, Sgs1/Dna2 serves as a back-up resection system after prolonged failure to use Exo1. The same might apply to MRX-Sae2 itself, in which case several iterations of resection of small stretches of DNA would be required; such an action could explain the much lower resection rate observed in the *exo1* mutant.

We further found that a fluorescently labeled genomic locus 3.4 kb distant from the HO cut site, marked by INT2-GFP, did not disappear in wild type cells. The length of the resected ssDNA tracts in HR-proficient cells has been reported to vary by others. It depended on the availability and location of the homologous template and was correlated with the kinetics of repair. In meiotic cells, the average length of ssDNA formed is 850 nt, whereas 2–4 kb ssDNA tails are formed during mitotic repair between chromosome homologs [60], [61]. We think it likely that the Ku-dependent delay in the onset of resection provides time for recruitment of the HR proteins that normally curtail resection and direct the generated ssDNA towards homology search. The absence of Ku allows resection often to escape this control. Appropriate placement of INT markers should allow the extent of resection to be addressed in more detail in future work.

We conclude with a note concerning the new visualization tool that enabled us to obtain these results. The ParB-INT visualization system allows direct kinetic measurements on single cells, avoiding interference with the process under study. These features should make the system widely applicable to the study of fine-scale chromosome positioning and dynamics in contexts beyond the repair process studied here, such as gene expression, replication and recombination.

## Materials and Methods

### Basis of the ParB-INT *in vivo* DNA labelling system

The system exploits the properties of proteins of the ParB family, whose function is to ensure mitotic stability of bacterial replicons through binding to sites (*parS*) to form a primitive kinetochore. ParB proteins interact with each other via a specific oligomerisation domain [62], [63]. Thus, a fluorescent ParB derivative initially binds to a small set of *parS* sites then recruits further ParB molecules which, by non-specific, relatively weak DNA binding, expand the complex to become a fluorescent focus. The same principle underlies use of the P1 plasmid ParB/*parS* pair as a generalized visualization tool [64], the difference here being that the dependence of this system on IHF, a host factor, disqualifies it for use in eukaryotes. Because nearly all the ParB protein is bound in a metastable fashion to DNA flanking the *parS* sites, it is readily displaced by transcription or other DNA-based processes while remaining available for rebinding to restore the fluorescent focus. Thus the insertion does not alter the dynamics of chromatin, its transcriptional status or its sensitivity to DNA damage. In addition, the greatly reduced size (<1 kb) of the INT sequence containing a reduced number of *parS* binding-sites facilitates targeted integration and stability of binding sites in bacteria, yeast and mammalian cells. We describe here the use of two ParB-INT variants, 1 and 2, based on the *B.cenocepacia* J2315 ParB/*parS* clusters of chromosome 2 and 3, respectively.

### Cloning of the B.cenocepacia J2315 *ParB* and *parS* (ParB and INT) sequences

Clusters of four *parS*c2 sites and four *parS*c3 sites were obtained by PCR as fragments representing base-pairs 1431–2453 of *B.cenocepacia* J2315 chromosome 2 and 3423–4585 of chromosome 3 (http://www.sanger.ac.uk/resources/downloads/bacteria/burkholderia-cenocepacia.html). The c2 and c3 fragments were first inserted into the ApaI-HpaI and ApaI-HindIII intervals, respectively, of the vector pMMB206 [65], then excised as AscI-MscI and BasaBI-HindIII fragments and inserted into the AscI-SmaI and HindIII-SmaI intervals of pAG60. The BglII-SpeI fragments of pAG25 and pAG32 [66] carrying genes for resistance to nourseothricin (*Nat*) and hygromycin (*Hyg*) respectively were inserted next to the *parS* segments in the pAG60 derivatives, yielding pFG2 and pFG4. The gene fusions *ParB*c2::mCherry and *ParB*c3::eGFP were amplified by PCR from plasmids pMLBADcat-*ParB*c2::mCherry and pMLBADcat-*ParB*c3::eGFP and inserted between the BamHI and NotI sites of pCM189 and pCM184 respectively to give pCM189-ParB1::mCherry and pCM184-ParB2::eGFP. These plasmids were used for construction of yeast strains, as described below.

### Yeast strains

Strains, plasmids and oligonucleotides are listed in supplementary tables S1 and S2. The base strain, YHS19, was derived from BMA64-1B (*ura3-1*, *trp1-Δ2*, *ade2-1* (ochre), *leu2-3*, *112*, *his3-11,15*, *can1-100* (ochre), *MAT*α) as follows. INT1 and INT2 cassettes were amplified by PCR from plasmids pFG2 and pFG4 respectively, using recombination primers Y alpha1 IntParSantisens FW and Y alpha1 IntParSantisens RW for insertion in the Yα1 region, and Mat Int 197 kb ParSFwandMatInt 197 kb ParSRw for insertion at 197 kb on chromosome III. BMA64-1B and JKM139 cells were transformed with the INT fragments and selected for resistance to nourseothricin or hygromycin [67]. Correct integration was verified by PCR using primers MAT101/Tef-Pro-Rw-verif and MAT5′-IT-F/Tef-Pro-Rw-verif for the Yα1 and 197 kb sites respectively. The INT derivatives were transformed as appropriate with pCM189-ParB1-mCherry with selection for Ura+ and pCM184-ParB2-GFP with selection for Trp+. The INT-ParB strains were transformed with plasmid pJH727 (pGal-HO), kindly provided by J. Haber. *yKu70* (YHS26) and *exo1* (YHS28) mutant strains were derived from YHS19 by transformation with the SpHis5 cassette amplified from pUG27 using His5-dyKu70-F/His5-dyKu70-R f and His5-dExo1-F/His5-dExo1-R respectively and verified by PCR on genomic DNA using dyKu70-His-Verif_F/dyKu70-His-Verif_R and dexo1-His-Verif_F/dexo1-His-Verif_R.

### Growth conditions

The medium used was SC-Leu with uracil and tryptophan omitted as appropriate for selecting plasmid maintenance. For microscopy, cells were grown overnight at 30°C with shaking in SC medium with 2% raffinose until reaching ∼5×10^7^ cells/ml, at which time galactose was added to 2% final and the cells directly processed for imaging.

### Microscopy

Time lapse experiments were performed using an Andor Revolution Nipkow-disk confocal system installed on an Olympus inverted microscope (IX81 S1F-3), featuring a YOKOGAWA CSU22 confocal spinning disk unit, a cooled Andor EMCCD camera (iXon^EM^ +DU888) to provide quantum efficiency (90%) and pixels at 13 µm×13 µm, an Olympus 100× fluorescence microscope oil objective (PlanSApo 1,40 oil immersion6) and an E-625 PZT Servo piezo. We excited the fluorophores with single diode pumped solid-state laser lines (DPSSL), GFP fluorescence at 488 nm (∼25 mW) and mCherry fluorescence at 561 nm (∼25 mW). We collected green and red fluorescence using a Semrock bi-bandpass emission filter (Em01-R488/568-15). Pixel size was 65 nm. EM gain of the EM-CCD camera was set to 300 for GFP and mCherry (pre-EM gain 5.20). Temperature was maintained at 30°C using a thermostated heater in an insulated box (Life Imaging Services). The system was controlled using Andor Revolution IQ software (version 2.0). For dual color Cherry and GFP imaging, 3D stacks of 36 planes over 7 µm (0.2 µm Z-step) were obtained at 400 ms and 200 ms acquisition time for mCherry and GFP respectively. Time lapse analysis of GFP foci was performed in 2D, acquiring stacks of 50 frames of 200 ms following HO induction. Stacks were acquired at 2 and 5 min intervals for *yku* and wt strains respectively starting at 8 minutes after HO induction. Controls were done in the overnight growth medium without addition of galactose.

### ChIP assays

ChIP analyses were performed as described previously [68] with minor modifications for yeast cells. Briefly, overnight cultures of untagged and INT-tagged strains were diluted an OD_600_ of 0.1 to in 150 ml of medium without tryptophan and grown to an OD_600_ of 1. Cells were fixed in 1% paraformaldehyde final for 15 min at room temperature with gentle shaking. Paraformaldehyde treatment was quenched by adding glycine to 125 mM. Five minutes later the cells were spun at 3000 rpm for 3 minutes at 4°C, washed twice with 10 ml of ice cold PBS and resuspended in 1 ml of ice cold PBS. 0.5 ml of FA lysis buffer was added and the cells transferred to a Lobind screw cap tube containing 0.5 ml of beads [69]. Cells were lysed by applying two 20-second pulses, separated by a 1 min pause, using a Bertin technology Precellys 24 (programme 3). The chromatin fraction was resuspended in nucleus lysis buffer and sonicated to generate DNA fragments of <500 bp. 500 µg of total DNA were subjected to immunoprecipitation using antibodies against GFP (1814860, Roche), phosphorylated H2A.X (39272, Active Motif), or H3 (ab1791, ABCAM), with HA antibody (H6908, Sigma) as a negative control. The precipitated DNA was amplified by real-time PCR, with primer sets designed to amplify the targeted sequences. The primers used in q-PCR are listed in table S2.

### Particle tracking, MSD calculation and fluorescence quantification

Particle tracking experiments and MSD calculations were carried out as described previously, [70] with modifications of the Image J Particle Detector and Tracker plugin to the following settings: Radius = 4, CutOff = 0, percentile = 0,1, displacement 10. Only tracks of more than 15 consecutive frames were scored. Diffusion coefficients (D) were calculated from the slope of the average MSD curve at the first 2 seconds. D of the non-induced, uncut locus was set to 100%. Intensities of the fluorescent foci were obtained with the Nikon NIS 3.2 AR element program using the intensity quantification line tool. Intensities of the pixels crossing the fluorescent focus were summed and normalized to the fluorescence intensity of the same number of pixels in a background region that does not contain the focus. The resulting fluorescence intensity ratio was normalized to 100% at t0 and followed over time. Fluctuations denote intracellular variations in fluorescence of either the background or the focus over time.

## Supporting Information

Table S1 Yeast strains.
(DOCX)

Table S2 Plasmids and oligos.
(DOCX)

Video S1 Time-lapse imaging of the fluorescent foci in a wt yeast cell. Left: INT1-mCherry focus disappears due to resection. Center: Persisting GFP focus. Right: Merge. First frame: 10 minutes after galactose addition, wait between 2 frames: 2 minutes. Scale bar: 2 µm.
(WMV)

Video S2 Persistence of the INT1-mCherry focus in the absence of DSB. Continuous live 2D stream showing the INT1/ParB1-mCherry fluorescent focus that persists in the absence of galactose (uncut condition).
(AVI)

Video S3 Persistence of the INT1 mCherry focus in the absence of DSB. Time-lapse movie of the INT1 mCherry focus extracted from 3D stacks. Time in minutes is indicated on the top right. Scale bar: 2 µm.
(AVI)

Video S4 Example of the GFP live stream acquisitions used for INT2 tracking. Scale bar: 2 µm. WT, uncut.
(AVI)

Video S5 Example of the GFP live stream acquisitions used for INT2 tracking. Scale bar: 2 µm. Cut & resected INT1.
(AVI)

Video S6 Example of the GFP live stream acquisitions used for INT2 tracking. Scale bar: 2 µm. *yku70* uncut.
(AVI)

Video S7 Example of the GFP live stream acquisitions used for INT2 tracking. Scale bar: 2 µm. *yku70* cut & resected INT1.
(AVI)
