# Supplementary material for: DNA Dynamics during Early Double-Strand Break Processing Revealed by Non-Intrusive Imaging of Living Cells
Source: PLoS Genet. 2014 Mar 13;10(3):e1004187. doi: 10.1371/journal.pgen.1004187 (PMC3952824; doi:10.1371/journal.pgen.1004187)
Supplement: Table S1 — Yeast strains. (DOCX) [file pgen.1004187.s001.docx]

| **Strain** | **Genotype** | **Plasmids** | **Source** | |  |
| --- | --- | --- | --- | --- | --- |
| BMA64-1B | *MAT*α *; ura3-52; trp1Δ 2; leu2-3,112; his3-11; ade2-1; can1-100* |  | [[1](#_ENREF_71)] | |  |
| YHS19 | *MAT*α *; ura3-52; trp1Δ 2; leu2-3,112; his3-11; ade2-1; can1-100 ; NatR-INT1 in Yalpha-1, INT2-HygR+* at 197kb on chromosome III | pCM184-ParB2-GFP; pCM189-ParB1-mCh; pJH727 | This work | |  |
| YHS26 | *MAT*α*; ura3-52; trp1Δ 2; leu2-3,112; his3-11; ade2-1; can1-100 ; NatR-INT1 in Yalpha-1, INT2-HygR+* at 197kb on chromosome III*; yku70::SpHis5* | pCM184-ParB2-GFP; pCM189-ParB1-mCh; pJH727 | This work | |  |
| YHS28 | *MAT*α*; ura3-52; trp1Δ 2; leu2-3,112; his3-11; ade2-1; can1-100 ; NatR-INT1 in Yalpha-1, INT2-HygR*+ at 197kb on chromosome III*; exo1::SpHis5* | pCM184-ParB2-GFP; pCM189-ParB1-mCh; pJH727 | This work | |  |
| YAN01 | *MAT*α*; ura3-52; trp1Δ 2; leu2-3,112; his3-11; ade2-1; can1-100 ; NatR-INT1 in Yalpha-1, INT2-HygR*+ at 197kb on chromosome III*; mre11::SpHis5* | pCM184-ParB2-GFP; pCM189-ParB1-mCh; pJH727 | This work | |  |
| YCP01 | *MAT*α *; ura3-52; trp1Δ 2; leu2-3,112; his3-11; ade2-1; can1-100 ; INT2-NatR+* at 197kb on chromosome III | pCM184-ParB2-GFP; pCM189-ParB1-mCh; pJH727 | This work | |  |
| YCP00 | *MAT*α *; ura3-52; trp1Δ 2; leu2-3,112; his3-11; ade2-1; can1-100 ;* | pCM184-ParB2-GFP; pCM189-ParB1-mCh; pJH727 | This work | |  |
| YHS46 | *MAT*α *; ura3-52; trp1Δ 2; leu2-3,112; his3-11; ade2; can1-100 ; NatR-INT1 in Yalpha-1, INT2-HygR+* at 197kb on chromosome III *dHML; dHMR; ade3::GAL::HO*  *GalHO integrated* | pCM184-ParB2-GFP; pCM189-ParB1-mCh; | | This work | |

*1. Baudin-Baillieu A, Guillemet E, Cullin C, Lacroute F (1997) Construction of a yeast strain deleted for the TRP1 promoter and coding region that enhances the efficiency of the polymerase chain reaction-disruption method. Yeast 13: 353-356.*
