# Supplementary material for: DNA Dynamics during Early Double-Strand Break Processing Revealed by Non-Intrusive Imaging of Living Cells
Source: PLoS Genet. 2014 Mar 13;10(3):e1004187. doi: 10.1371/journal.pgen.1004187 (PMC3952824; doi:10.1371/journal.pgen.1004187)
Supplement: Table S2 — Plasmids and oligos. (DOCX) [file pgen.1004187.s002.docx]

| **Plasmid** | **Vector; insert** | | **Reference** |
| --- | --- | --- | --- |
| pFG2 | pDAG512; Int1::NatR | | This work |
| pFG4 | pDAG514; Int2::HygR | | This work |
| pCM189-ParB1-mCh | pCM189; ParB1::mCherry | | This work |
| pCM184-ParB2-GFP | pCM184; ParB2::GFP | | This work |
| pJH727 | pGAL::HO | | [[1](#_ENREF_72)] |
| pUG27 | pSpHis5 | | [[2](#_ENREF_73)] (euroscarf) |
| **Oligo** | | **Sequence** | |
| Y alpha1 Int ParSantisens FW | | 5’-TCAGCGAGCAGAGAAGACAAGACATTTTGTTTTACACCGGAGCCAAACTGGTGACACTATAGAACGCGGCCGCCA-3’ | |
| Y alpha1 Int ParSantisens RW | | 5'-CTTCAAAGAAATATTTAAACTCATTTATGGCTTTTAGAGCATATTACTCATATAGGGAGACCGGCAGATCCGCGG-3’ | |
| Mat Int 197kb ParS Fw | | 5’-TAGCCGCCTTTACCGTAGTTTTGCTGCACCTTTATCTGAGAGCTGACTGCGTGACACTATAGAACGCGGCCGCCA-3’ | |
| Mat Int 197kb ParS Rw | | 5’-GAAAAGTAATCCGATGCATTGAAAAATTATTACCAGAAAACAGTGTTTCATATAGGGAGACCGGCAGATCCGCGG-3’ | |
| MAT101 | | AGTCACATCAAGATCGTTTATGG | |
| Tef-Pro-Rw-verif | | ATACATGGGGATGTATGGGC | |
| MAT5’-IT-F | | CAT TCA GGG ACA GCG TTG TAT | |
| His5-dyKu70-R | | CTACCAAATATTGTATGTAACGTTATAGATATGAAGGATTTCAATCGTCTaatacgactcactatagggag | |
| His5-dyKu70-F | | AGCTATGATTTGTTAAGTGACTCTAAGCCTGATTTTAAAACGGGAATATTcacatacgatttaggtgacac | |
| dyKu70-His-Verif_F | | ACAATTATCAAATTATTCACCCAATATTCAGTATAATCC | |
| His5-dExo1-F | | ACCACATTAAAATAAAAGGAGCTCGAAAAAACTGAAAGGCGTAGAAAGGAcacatacgatttaggtgacac | |
| His5-dExo1-R | | TTTTCATTTGAAAAATATACCTCCGATATGAAACGTGCAGTACTTAACTTaatacgactcactatagggag | |
| dExo1-His-Verif_F | | AAATCACTGGAAGACGAATTTGACGGATTTTTGGCTTAATA | |
| 1 up Fw | | TTTCGATGAAGATGGTTCTCCT | |
| 1 up Rw | | AAATACAACGCTGTCCCTGAAT | |
| 2 up Fw | | ACACGTCAACGTCATCATCTTC | |
| 2 up Rw | | AATTCAAGCATTGCCTCCTTTA | |
| 3 up Fw | | TTGGCTTAGGCAATGGATACTT | |
| 3 up Rw | | ACCACATTGTTTGGGAAAGAAG | |
| 4 up Fw | | CAGCGGATCTAGTAAGGGTGTT | |
| 4 up Rw | | AAAGTTTTGGCATCGTGGTTAT | |
| 5 up Fw | | TGTCCACTTCCTCTTCTTCAGAG | |
| 5 up Rw | | GGAAGAAGTGCCACCATAAGAG | |
| 6 up Fw | | AGACCTCGATCAATGGATGATT | |
| 6 up Rw | | TGATTATCAAATGGTGGTCTGG | |
| 1 dw Fw | | AACTGACCACAAATCCCCTATC | |
| 1 dw Rw | | ATATCGATTCCTCCGAGTGCTA | |
| 2 dw Fw | | GCGCCCCTGTAGAGAAATATAA | |
| 2 dw Rw | | GGTAAGCCGTGTCGTCAAGAGT | |
| 3 dw Fw | | GAGCAGGCGCTCTACATGA | |
| 3 dw Rw | | AGATGATGTCGAGGCGAAAA | |
| 4 dw Fw | | AACGTTTCCTATGCACAATCTT | |
| 4 dw Rw | | GCTAAGGTTCCAAGGCTTACCT | |
| 5 dw Fw | | GTAAACAAAACAAGGGCATCCT | |
| 5 dw Rw | | TTGCATAAAGTGGGTGCTTATG | |
| 6 dw Fw | | AAGTCAAACAGTTCCAATGACA | |
| 6 dw Rw | | TGACTGGTCGTACACATACAACA | |
| Fw-ParSc2+-5’-antisens1 | | CATAAACCGATCGTCCGAAT | |
| Rw-Z1-1 | | ACTGTTGCGCGAAGTAGTCC | |
| Fw-ParSc2+-5’-antisens2 | | AACATCCACCGCTCGTTTAT | |
| Rw-Z1-2 | | TCTTCCCAATATCCGTCACC | |
| Fw-internal-ParSc2+1 500/700 | | TCAGGTGCGTAGATCACGAG | |
| Rw-internal-parSc2+1 | | ACATATGTCGACACGCAACG | |
| Fw-internal-ParSc2+2 700/900 | | GTGTTCCGCAATCGCATAC | |
| Fw-internal-ParSc2+2 700/900 Rw | | GCGTCGAAGAGTGGTTGTTT | |
| ChiP_h2A_tel6R_Fw | | ACGTTTAGCTGAGTTTAACGGTG | |
| ChiP_h2A_tel6R_Rw | | CATGACCAGTCCCTCATTTCCATC | |

References

*1. White CI, Haber JE (1990) Intermediates of recombination during mating type switching in Saccharomyces cerevisiae. Embo J 9: 663-673.*

*2. Gueldener U, Heinisch J, Koehler GJ, Voss D, Hegemann JH (2002) A second set of loxP marker cassettes for Cre-mediated multiple gene knockouts in budding yeast. Nucleic Acids Res 30: e23.*
